# Nurses’ application of the components of family nursing conversations in home health care: a qualitative content analysis

**DOI:** 10.1111/scs.12731

**Published:** 2019-06-28

**Authors:** Susanne Broekema, Wolter Paans, Petrie F. Roodbol, Marie Louise A. Luttik

**Affiliations:** ^1^ Research Group Nursing Diagnostics Hanze University of Applied Sciences Groningen the Netherlands; ^2^ Faculty of Medical Sciences University of Groningen Groningen the Netherlands

**Keywords:** family caregivers, family nursing conversation, family nursing intervention, family systems nursing, home care, nursing, qualitative content analysis

## Abstract

**Aim:**

The purpose of this study was to describe how nurses apply the components of family nursing conversations in their home healthcare practice.

**Method:**

A qualitative content analysis with a deductive approach was conducted. Home healthcare nurses conducted family nursing conversations with families from their practice. Families were selected based on three nursing diagnoses: risk of caregiver role strain, caregiver role strain or interrupted family processes. Nurses audio‐recorded each conversation and completed a written reflection form afterwards. Transcripts of the audio‐recorded conversations were analysed in Atlas.ti 8.0 to come to descriptions of how nurses applied each component. Nurses’ reflections on their application were integrated in the descriptions.

**Results:**

A total of 17 conversations were audio‐recorded. The application of each component was described as well as nurses’ reflections on their application. Nurses altered or omitted components due to their clinical judgment of families’ needs in specific situations, due to needs for adjustment of components in the transfer from theory to practice or due to limited skill or self‐confidence.

**Conclusion:**

All of the components were applied in a cohesive manner. Nurses’ application of the components demonstrates that clinical judgment is important in applying them. Further training or experience may be required to optimise nurses’ skill and self‐confidence in applying the components. This study demonstrates the applicability of the family nursing conversations components in home health care, allowing exploration of the working mechanisms and benefits of family nursing conversations for families involved in long‐term caregiving in future studies.

## Introduction

Illness of a family member impacts the entire family. Family relationships, roles and tasks as well as activities and communication may change 1. In situations of severe stress, such as illness, resilient families will find effective ways for positive adaptation, whereas the confrontation with an illness could result in crisis for those that are less resilient 2, 3. Caring for a family member might bring about positive experiences 4 and have favourable effects on the health and well‐being of family members 5, 6. However, those family members that provide intensive care are especially at risk for caregiver burden with negative consequences for their health and work participation 7, 8, 9, 10, 11 as well as for the quality of the care they provide 12. Support from professional caregivers seems to be important for preventing or decreasing family caregiver burden 13. A recent integrative review found that, in addition to such supportive care needs, family caregivers also consider collaboration with home care nurses important in caring for the patient 14, 15.

The theory of family systems nursing emphasises that nurses should approach families rather than only the patients as the unit of care as families are always impacted by illness 16. An important intervention within family systems nursing is the Family Health Conversation that was developed in Sweden 17. These conversations have been described in terms of 12 well‐defined core components 18. Such a clear and specific description of an intervention is beneficial for educating professionals and is likely to increase intervention integrity 19. The Family Health Conversation model is an intervention that typically consists of three conversations over a period of six to ten weeks that are intended to solve problems that negatively affect family health 17. The intervention is concluded by sending the family a closing letter with nurses’ reflections. From studies regarding Family Health Conversations, it appears that the intervention was delivered by nurses that were not involved in daily care for the patient 18, 20, 21.

Within the current study, a family nursing intervention that is intended to be conducted on a regular basis as part of routine nursing care is described. Incorporation into routine nursing care is considered important to facilitate family–professional collaboration in long‐term care situations. Therefore, this intervention, the family nursing conversation, is to be conducted by the nurse that also provides and coordinates the regular care for the patient. Family nursing conversations are aimed at fostering family resilience, facilitating collaboration between family members and professional caregivers, and preventing or decreasing caregiver burden. The family resilience framework identifies three domains of key family processes that professionals can focus on in order to foster family resilience 2, 22, 23. First, resilient families hold beliefs that are optimistic and hopeful and that allow them to give meaning and purpose to the adverse situation. Second, in terms of organisation, resilient families are flexible and able to adapt to a changed situation; they are connected, support each other and can tolerate differences; and they have access to resources. Third, communication of information and emotions in resilient families is clear and open, and families collaboratively solve problems and make shared decisions.

The Family Health Conversation components 18 have been adapted to allow incorporation into routine nursing care and achievement of the aims of family nursing conversations, especially family resilience processes, as shown in Table 1. All of the components are to be applied in relation to the care situation. More widespread or complex individual or family issues may be identified but are only discussed further when they are relevant to the care situation and are within the nurse’s expertise. When this is not the case, nurses will refer the family to appropriate professionals and focus the family nursing conversation on the care situation.

**Table 1:** The components of family nursing conversations in relation to the family resilience processes that the components are intended to contribute to

Components of family nursing conversations (adapted from Östlund and colleagues 18)		Family resilience processes and family functioning domain 2, 22
1	Jointly reflecting with the family on expectations of the conversation, and jointly setting the goal for the conversation.	Clarity^a^ Collaborative problem‐solving^a^ Positive outlook^c^
2	Getting to know each other; who is present and who is absent.	Connectedness^b^ Social and economic resources^b^
3	Exploring the family structure and finding out who is part of the family by making and discussing the genogram with the family.	Connectedness^b^ Social and economic resources^b^
4	Exploring relationships within the family and relationships between the family and other people and organisations by making and discussing the ecomap with the family.	Connectedness^b^ Social and economic resources^b^
5	Inviting each family member to share their story and narrate expectations, needs, and emotions related to the care situation.	Clarity^a^ Open emotional expression^a^ Connectedness^b^ Make meaning of adversity^c^
6	Formulating a shared question or problem regarding the care situation.	Collaborative problem‐solving^a^ Connectedness^b^
7	Acknowledging painful experiences and events and related emotions.	Open emotional expression^a^ Connectedness^b^
8	Giving commendations about family strengths, competencies and resources.	Positive outlook^c^
9	Stimulating open communication between family members, also about difficult topics	Clarity^a^ Open emotional expression^a^ Connectedness^b^
10	Signalling and discussing family members’ beliefs related to the care situation. Challenge constraining beliefs and support facilitating beliefs.	Flexibility^b^ Positive outlook^c^ Make meaning of adversity^c^
11	Summarising the central issues that have been raised and pursued in the conversation.	Clarity^a^ Make meaning of adversity^c^
12	Setting joint goals and agreements for the care situation.	Collaborative problem‐solving^a^ Flexibility^b^

a Communication/problem‐solving.

b Organizational patterns.

c Belief systems.

### Aim

This study is part of a larger project in which family nursing conversations are developed, implemented and tested in nursing practice in the Netherlands, using the knowledge‐to‐action framework 24. As part of the implementation process, this framework emphasises the need to monitor how knowledge is actually used when it is applied in practice, in order to further adapt it to the local context and ultimately evaluate its effects. As this is the first time the family nursing conversation components are transferred from theory to home healthcare practice, the aim of this study was to describe how nurses’ apply each of the described components in their family nursing conversations in home health care.

## Methods

### Design

This study was conducted using a qualitative content analysis 25 with a deductive approach. The units of analysis were the transcripts of audio‐recorded family nursing conversations and nurses’ reflection forms about these conversations.

### Participants

Ten home healthcare nurses from three home healthcare organisations in the northern part of the Netherlands conducted the family nursing conversations. These nurses also coordinated and participated in the routine care for the patients. Nurses were all female with a mean age of 47 (± 9) years and, on average, 13.5 (± 12) years of work experience. All of the nurses had recently received a six‐day educational intervention on family systems nursing and family nursing conversations as described elsewhere 26. The family nursing conversation components were a part of the educational intervention.

### Data collection

In the three months following the educational intervention (January – April 2017), participating nurses were asked to organise and conduct family nursing conversations with three families from their daily practice. In accordance with the aims of these conversations, nurses selected families with challenged family functioning or with family caregivers at risk for overburden. The selection, therefore, was based on the following NANDA‐I nursing diagnoses 27: 1) risk of caregiver role strain; 2) caregiver role strain; and 3) interrupted family processes. The nurses individually conducted the family nursing conversations. When the family agreed, the nurse audio‐recorded the conversation and subsequently completed a written reflection form on which she reflected on her application of each of the components.

### Ethical considerations

Approval of the research project of which this study was a part was waived by the medical ethical committee of the university (M15.182392/METc2015.463), as the study does not fall under the Dutch Medical Research Involving Human Subjects Acts. All of the participants to the family nursing conversations received verbal and written information about the study’s purpose and procedures. Participants were informed that their data would be treated confidentially, and all names, addresses and other identifiable personal details would be removed from the transcripts and in the analysis. The conversations were audio‐recorded when all of the participants in the conversations provided their written informed consent. Participants could refuse or withdraw their consent at any time without consequences for the received nursing care. The audio recordings and transcripts were stored without identifiable information.

### Data analysis

All of the audio‐recorded family nursing conversations were transcribed verbatim and analysed using Atlas.ti 8.0. Before analysing a transcript, the researcher read through the transcript while listening to the audio recording, both to check the transcript’s accuracy and to gain understanding of the complete conversation. The analysis focused on the manifest content of the transcripts and the reflection forms.

Analysis occurred in three phases. During the first deductive phase, all occurrences of the 12 components in nurses’ contributions to the conversations were coded as such. First, four research assistants (fourth‐year bachelor nursing students) coded each conversation in pairs and then discussed their coding to reach consensus. Subsequently, the first author independently coded the conversations and compared the coding to that of the research assistants. Discrepancies were discussed and easily resolved. In the second phase, all text fragments within each component were read through multiple times in order to allow creating a description of the application of the components. Fragments were also read in the context of the complete conversation to describe how components were integrated. The first author formulated the descriptions and subsequently discussed and refined these with the other authors. In the final analysis phase, the outcomes of the first two phases were compared with the written reflections that nurses provided on their use of the components. Attention was paid to any clarifications or explanations that nurses provided in their reflections regarding their application of the components. Comments from the reflection forms were integrated into the descriptions when relevant to explain the application of the components.

## Results

Within the preset time period, a total of 17 family nursing conversations with 17 families were successfully audio‐recorded. An additional 15 conversations were conducted but were not audio‐recorded due to failing recorders (n = 7), families that declined permission for audio recording (n = 5) and nurses who felt uncomfortable asking permission for audio recording (n = 3). Each nurse delivered at least one audio‐recorded conversation. Saturation was reached, and quotes from all 17 conversations were considered for use in the descriptions. The 17 audio‐recorded conversations lasted 41 (± 16) minutes on average. For 13 of them, the nurse completed the reflection form. An overview of conversation participants is provided in Table 2.

**Table 2:** Participants in the family nursing conversations

Conversation no.	Nurse no.	Participants	Reason for receiving care
1	1	Patient; partner	Palliative phase
2	1	Patient; partner; sister	Palliative phase
3	2	Patient; partner	Impaired self‐reliance
4	2	Patient; partner; daughter; son‐in‐law	Dementia
5	3	Patient; partner	Chronic obstructive pulmonary disease
6	3	Patient; partner	Cardiovascular accident
7	4	Partner; neighbour; neighbour	Palliative phase, dementia
8	4	Patient; cousin	Impaired self‐reliance
9	5	Patient; patient; nurse	Cardiovascular accident; impaired self‐reliance
10	5	Patient; patient; nurse	Impaired self‐reliance
11	6	Patient; patient; daughter; daughter	Dementia
12	6	Patient; daughter; daughter	Dementia
13	6	Patient; son; son	Dementia
14	7	Daughter; granddaughter; community psychiatric nurse	Dementia
15	8	Patient; partner	Chronic obstructive pulmonary disease
16	9	Patient; partner; nurse	Impaired self‐reliance
17	10	Patient; partner; daughter	Palliative phase

### Description of the Results per family nursing conversation Component

*Component 1: Jointly reflecting with the family on expectations of the conversation and jointly setting the goal for the conversation.* The conversations typically began with an open question about the care situation, which was sometimes preceded by a statement about the nurse’s expectation or goal for the conversation. Such a statement is illustrated in the following example:

Nurse: You already asked me why I am here and the boys are here. I said that it is important to talk to each other about how things are going and how we see the future with you. […] I have had some telephone contact with your sons but have never seen them. And sometimes it is quite good to sit together and share everything together. To see how things go and what should happen when things go less well.

[Conversation 13]

In the reflection forms, a number of nurses mentioned that they considered it not appropriate to ask the family about expectations and goals for a conversation that had been initiated by the nurse. Other nurses commented that, in some situations, it was difficult to step back from the urgency of the care situation and discuss the context or conditions for the conversation. Finally, several nurses reported that they had forgotten to apply this component.

*Component 2: Getting to know each other; who is present and who is absent.* In conversations in which participants did not yet know each other, this component was combined with component 3; discussing the family structure and making a genogram. This combination automatically allowed for giving attention to family members who were absent, such as in the following quote:

Nurse: All right, I have the participants’ names. I would very much like to know who else is part of the network of Mr. and Mrs. X. I understand there is another daughter?

[Conversation 4]

*Component 3: Exploring the family structure and finding out who is part of the family by making and discussing the genogram with the family.* Two variants of this component occurred. In some conversations, the nurse explored the family structure and made a genogram during the conversation, and in others, the family structure was explored without making a genogram. In the reflection forms, these nurses explained that they did not feel comfortable drawing during the conversation. Some nurses made the genogram afterwards. The exploration of the family structure was usually led by the nurse with specific questions about family members. In cases in which a genogram was made, this was usually introduced informally:

Nurse: May I ask, how many children do you have?

Partner: Two.

Nurse: Two daughters?

Patient: Yes.

Nurse: I’ll draw along, that’s called a genogram. So I’ll write down Mrs. and Mr. X, two daughters. Are both of your daughters married, or do they have partners?

[Conversation 16]

Differences existed in the extensiveness of the exploration, for example, the number of family members and individual characteristics that were included. When the family structure was not explored, nurses reported that they either already had sufficient knowledge about the family structure or that they prioritized other topics.

*Component 4: Exploring relationships within the family and relationships between the family and other people and organisations by making and discussing the ecomap with the family* *.* For this component, again, two variants occurred: an exploration of the family’s relationships by making an ecomap, or a verbal exploration of the family’s relationships with an ecomap sometimes being made afterwards. The exploration of relationships within the family was usually combined with component 3, exploring the family structure. Nurses decided what aspects of family relationships were discussed, such as the quality of the relationship: ‘Good contact with her?’, or the support that is or could be provided: ‘You get a lot of support from them?’. Family members were subsequently openly invited to share others who were important to them which afforded an exploration of relationships with people outside the family and with organisations:

Nurse: And do you have friends or other networks that you would say are very important to you?

[Conversation 3]

*Component 5: Inviting each family member to share their story and narrate expectations, needs and emotions related to the care situation.* Nurses primarily asked open questions in order to elicit stories and the subsequent emotions, needs and/or expectations. As family members narrated their stories, nurses encouraged them and suggested new topics. In some conversations, all family members were invited individually to share their story. In others, the nurses invited the family as a whole and sometimes the two approaches were combined. Family members that were less visible in the conversation were usually actively involved by the nurse. In the reflection forms, nurses indeed stressed their efforts to provide every family member the opportunity to share their story. This is evident in the following successive actions from the nurse; family members’ responses are left out.

Nurse [to patient]: How do you feel about that [the professional care]?

Nurse [to daughter 1]: How do you think things are going?

Patient: […]

Nurse [to patient]: But, if you don’t mind, I would like to ask your daughter. Is that okay with you?

[Conversation 12]

*Component 6: Formulating a shared question or problem regarding the care situation.* This component typically occurred after all of the family members had shared their stories. The nurse then extracted a question or problem shared by most or all of the participants. These shared questions or problems tended to be related to the care situation as a whole, such as caregiver overburden, or what care and support is needed to allow a patient to safely live at home. The following quote illustrates an example of such a shared question and problem that is based on the stories that were shared:

Nurse: Sometimes it is a good idea to have other people monitor, see how we can support you, because it is a very delicate balance.

Patient: Yes.

Nurse: And your husband, because he is seriously ill… To see how we can keep you going for as long as possible.

Neighbor 1: Yes.

Neighbor 2: Exactly.

[Conversation 7]

*Component 7: Acknowledging painful experiences and events and related emotions.* Nurses acknowledged painful experiences and emotions by naming or repeating them, by legitimizing them or showing understanding, or by asking questions about them. Nurses focused on the experience or event, on the related emotions, or on both:

Nurse: That was quite distressing for you as well because you are in a process of illness yourself, and then this in addition [wife’s leg fracture]... You worry about that quite a bit, I can understand that.

[Conversation 1]

Some nurses explained in the reflection forms that they did not acknowledge a painful topic when it had been previously discussed multiple times during the conversation or when the nurse knew from prior experience that it would have overly upset the participants.

*Component 8: Giving commendations about family strengths, competencies and resources.* Nurses pointed out family strengths, competencies and resources themselves and affirmed positive aspects when they were mentioned by family members. Nurses paid careful attention to give a commendation at every available opportunity. Commendations took the form of statements, suggestions or questions that were integrated in the conversation naturally:

[Family and nurse laughing together].

Nurse: There seems to be quite a sense of humor here.

Partner: Yeah, we had that right from the beginning […]. You know, there is nothing you can do about it and you could stay inside all day, but that won’t make things any better.

Nurse: No, but still it is impressive that you are able to do this and just do it.

Partner: No, not everyone could do that, true.

[Conversation 6]

Family members tended to elaborate on strengths, competencies and resources that the nurse commended them for, thus providing the nurse with new opportunities for commendations.

*Component 9: Stimulating open communication between family members, also about difficult topics.* Nurses stimulated open communication about difficult topics by introducing these topics and asking family members about their perspective. Over the course of the conversation, a shift occurred from only communication between the family and the nurse to also communication between family members, through intervention by the nurse:

Nurse: Do you understand why your daughter says she worries about you?

Partner: Yes, well, we have of course had this whole situation with the accident and all, and she had to help […]

Daughter: Yes, that is one reason, but also […]

[Conversation 17]

In other conversations, there are no clear indications in the transcripts as to how this shift occurred. In the reflection forms, some nurses explained that they encouraged open communication among family members by leaning backwards a bit, both physically and verbally, thus giving space to the family.

*Component 10: Signalling and discussing family members’ beliefs related to the care situation* *. Challenge constraining beliefs and support facilitating beliefs.* Nurses reacted to beliefs that family members spontaneously mentioned, and sometimes actively elicited family members’ beliefs. Support of facilitative beliefs occurred with a brief confirmation (e.g. ‘That’s true’), by reformulating or repeating the belief, or by sharing opinions or experiences that affirmed the belief. This usually occurred in the flow of the conversation. Challenging constraining beliefs involved explicit discussion of beliefs and offering alternative beliefs:

Nurse: But what do you think about it, now that we are discussing that we expect a larger contribution from the children? Is that painful?

Patient: No, no. I don’t want to put too much pressure on the children. Because that happened to me in the past, an awful lot of pressure. Terrible, that was just abnormal.

Nurse: Yes, indeed. I can understand that very well, but you should not overdo it by not asking any help at all. Because it would be good for your children to know that you do really need help now. As long as you don’t indicate that, they won’t see the seriousness of the situation. They will think: oh well, mum can manage.

[Conversation 7]

In the reflection forms, some nurses mentioned that they signalled constraining beliefs but either did not want to confront the family at this stage of the contact or did not feel capable of challenging these beliefs. Supporting facilitating beliefs was considered to be easier.

*Component 11: Summarising the central issues that have been raised and pursued in the conversation* *.* Summaries occurred throughout the conversation. At the beginning, some nurses summarised previous conversations. During the conversation, brief summaries of a topic that had just been discussed were offered in order to mark the transition to a next topic. At the end of the conversation, sometimes all of the issues that were raised during the conversation were summarised, sometimes only the last topic and sometimes only the agreements that were made. In some situations, nurses actively invited the family to respond or contribute to the summary, as in the following quote. After each question or statement by the nurse, family members confirmed what was said or added information; these responses are omitted:

Nurse: We discussed the nursing home; you would like to go there, but not just yet.

Nurse: And, for now, things are okay at home.

Nurse: You are satisfied with the care you receive

Nurse: And how about you, madam? Is there anything you would like to add?

Nurse: Things are okay for now, is that right?

Nurse [to the daughters]: And you? Well, I already asked you.

[Conversation 11]

*Component 12: Setting joint goals and agreements for the care situation.* Nurses stated in the reflection forms that joint goal setting mostly occurred during component 6; formulating a shared question or problem. Agreements were made about professional and informal care and support for the patient and family members. Nurses provided information and suggestions about available care and support and discussed and decided with the family what options were most feasible. These agreements tended to be made throughout the conversation and were usually related to the shared question or problem. In the following conversation, for example, each agreement contributed to the patient living safely at home and simultaneously preventing overburden of the only family caregiver; his responses are removed:

Nurse: So the situation might even worsen a bit, but we need to stay ahead of that and alert each other about any changes we might see.

Nurse: And if she wants to stay at home, we have to organize that in the right way with the right means so that we can provide care.

Nurse: And don’t worry, there are many opportunities, such as an adjustable bed, a patient lift […]

Nurse: So usually the family should put a request in at the municipality but, in this case, I could do that with your permission and then contact you again.

[Conversation 8]

## Discussion

This study aimed to describe how home healthcare nurses applied family nursing conversation components during the first transfer of these components to nursing practice, as part of a larger implementation project. All components occurred in the conversations. Nurses typically introduced components implicitly by applying them, for example, asking a question about the family structure or inviting family members to share their story. Some components seemed more easy to apply than others, for example, ‘inviting family members to share their story’ and ’giving commendations about family strengths, competencies and resources’. Nurses described other components as more difficult, for example,’jointly reflecting with the family on expectations for the conversation and setting a joint goal’, and ‘signalling and challenging family members’ constraining beliefs related to the care situation’. The reflection forms revealed some lack of self‐confidence or skill. This will need to be resolved, possibly through gaining more experience, since in the Swedish study into the components of Family Health Conversations 18, the occurrence of some components already increased in the second and third conversation. In addition, in our earlier study evaluating the educational intervention 26, nurses indeed recommended additional experience rather than more education to improve their feelings of competence. Nevertheless, it may also be that additional educational needs will arise.

Components were applied in connection with each other. Nurses, for example, typically extracted the ‘shared question or problem regarding the care situation’ from ‘family members’ shared stories, expectations, needs and emotions’. Subsequently, the shared question was formulated as a goal to which ‘joint goals and agreements for the care situation’ were related. Moreover, nurses tended to apply a certain structure in the components: during the first four components, the nurse gathered specific information, using closed‐ended questions in accordance with communication theories 28, 29. Then starting with component 5, the family was encouraged to share and participate, through a larger number of open‐ended questions. In terms of family resilience processes, the focus was first on organisational patterns and shifted gradually to communication/collaborative problem‐solving and belief systems 2, 22.

Nurses adapted their application of the components in order to optimally serve the needs of the care situation and the family. An example is the decision to postpone the exploration of the family structure when a family is obviously preoccupied with an urgent issue in the care situation. These adaptations were generally well substantiated and based on nurses’ clinical judgment 30. A flexible approach rather than a strict protocol in applying the components may, therefore, be argued for. In future education, explicit attention to clinical reasoning and decision‐making in the context of family nursing conversations would be valuable for optimising the fit between the intervention and the family situation 31. Such a need for adaptation to the context in order to have optimal effect is one of the factors that defines an intervention as complex 32. Fidelity is traditionally defined as the degree to which intervention components are conducted as planned 33. In complex interventions, it may be more feasible to define fidelity as the degree to which the underlying function of components is achieved in practice 34. To assess and optimise nurses’ fidelity to the function of the components, it will be necessary to come to understand the working mechanisms of the family nursing conversations components in practice 35. This study provides an overview of the way nurses apply the components in family nursing conversations in their everyday home healthcare nursing with a heterogeneous sample of patients and families. As such, it demonstrates the real‐world applicability of the components in home healthcare nursing, by regular nurses that participated in a six‐day educational intervention 26.

This study does not allow statements about the effectiveness of the conversations with regard to the aims of decreasing family caregiver burden and achieving family resilience and family–nurse collaboration. However, the joint goals and agreements that were developed during the conversations were generally related to these aims. In addition, the components were applied in a way that family resilience processes 2, 22 were encouraged: family structure and social resources were discussed, a positive outlook was encouraged, and open communication and collaborative goal setting and problem‐solving occurred. Family resilience processes could be further encouraged through meta‐communication with the family about the purpose and expectations of the family nursing conversation at the beginning of the conversation. This first component was hardly present in the conversations that were analysed in this study. The component is, however, important for immediately alerting families that this conversation will be different from other contacts with healthcare professionals in that collaboration with the family, and therefore, the family’s contribution is crucial. By applying the component, family resilience processes could be encouraged from the beginning of the family nursing conversation.

### Limitations

First, visual recordings of the conversations in addition to the audio recordings would have allowed inclusion of the nonverbal strategies that nurses used to apply the components. Second, this study only provides insight into the application of the components by nurses who were relatively inexperienced in family nursing conversations. It is, therefore, not possible to disentangle needs for more experience from needs for further educational interventions. Thirdly, this qualitative study cannot be used to assess the overall quality of the conversations that were conducted, as it only describes the ways in which nurses applied the components and nurses’ reflections on their application. The results indicate that the needs of each specific care situation should be taken into account in order to evaluate the quality of a family nursing conversation; only assessing the degree to which a conversation includes all theoretical components does not suffice. Finally, despite and partly due to the heterogeneous sample, the relatively small sample size did not allow for exploration of the application of components in subgroups of patients, families or nurses. The study therefore provides limited insight in the reasons behind variation in nurses’ application of the components; insight is solely based on nurses’ reflection forms.

## Conclusion

Nurses applied the family nursing conversation components in a cohesive manner tailored to the care situation. Nurses’ application of the components demonstrates that the components can be applied in daily home healthcare nursing. It will be important to assess their applicability in other settings including hospital care, residential care and mental health care. Nurses’ clinical judgment was important to tailor the components to the needs of individual families. Future research is necessary to assess the effectiveness and working mechanisms of family nursing conversations according to the described components in fostering family resilience, preventing family caregiver burden and optimising collaboration between the family and professional caregivers.

## Conflict of interest

No conflict of interest has been declared by the author(s).

## Ethical considerations

Approval of the research project of which this study was a part was waived by the medical ethical committee of the university (M15.182392/METc2015.463), as the study does not fall under the Dutch Medical Research Involving Human Subjects Acts. All of the participants to the family nursing conversations received verbal and written information about the study’s purpose and procedures.

## Funding

This study was conducted with financial support of the Centre of Expertise Healthy Ageing of the Hanze University of Applied Sciences in Groningen, the Netherlands and the Dutch Health insurance company Menzis. The funding organisations had no involvement in the study design; in the collection, analysis and interpretation of data; in the writing of the report; or in the decision to submit the article for publication.
